# Accuracy of point-of-care multiorgan ultrasonography for the diagnosis of pulmonary embolism

**DOI:** 10.1186/2036-7902-6-S1-A25

**Published:** 2014-01-31

**Authors:** Peiman Nazerian, Simone Vanni, Giovanni Volpicelli, Chiara Gigli, Maurizio Zanobetti, Alessandro Lamorte, Andrea Fabbri, Stefano Grifoni

**Affiliations:** 1 Department of Emergency Medicine, Careggi University Hospital, Firenze, Italy; 2 Department of Emergency Medicine, San Luigi Gonzaga University Hospital, Torino, Italy; 3 Department of Emergency Medicine, Pierantoni Morgagni Hospital, Forlì, Italy

## Background

Presenting signs and symptoms of pulmonary embolism (PE) are non-specific, favoring a large use of second-line diagnostic tests such as multi-detector computed tomography pulmonary angiography (MCTPA), thus exposing patients to high-dose radiation and to potential serious complications.

## Objective

We investigated the diagnostic performance of multiorgan ultrasonography (lung, heart and leg veins ultrasonography) and if multiorgan ultrasonography combined to Wells score and D-dimer could safely reduce MCTPA tests.

## Patients and methods

Consecutive adult patients suspected of PE and with a Wells score >4 or a positive D-dimer were prospectively enrolled in three emergency departments. Final diagnosis was obtained with MCTPA. Multiorgan ultrasonography was performed before MCTPA and considered diagnostic for PE if one or more subpleural infarcts, right ventricular dilatation or deep vein thrombosis were detected. If multiorgan ultrasonography was negative for PE, an alternative ultrasonography diagnosis was searched for. Accuracies of each single-organ and multiorgan ultrasonography were calculated.

## Results

PE was diagnosed in 110 (30.8%) out of 357 enrolled patients. Multiorgan ultrasonography yielded a sensitivity of 90% and a specificity of 86.2%, lung ultrasonography of 60.9% and 95.9%, heart ultrasonography of 32.7% and 90.9% and vein ultrasonography of 52.7% and 97.6% respectively. Among the 132 (37%) patients with multiorgan ultrasonography negative for PE plus an alternative ultrasonographic diagnosis or plus a negative D-dimer, no patients had PE as final diagnosis.

## Conclusions

Multiorgan ultrasonography is more sensitive than single-organ ultrasonography, increases the accuracy of clinical pre-test probability estimation in patients with suspected PE and may safely reduce the MCTPA burden.

